# EBV gL-gH344-Ferritin Nanoparticle Vaccine Elicits Robust Immune Responses in Mice

**DOI:** 10.3390/v17060754

**Published:** 2025-05-26

**Authors:** Chenyu Li, Yuxi Cao, Qi Ma, Jing Yang, Xiaoguang Zhang, Hongxia Li, Ke Xu, Tao Jiang, Shuying Li, Yanzhe Hao, Xia Feng

**Affiliations:** 1 National Key Laboratory of Intelligent Tracking and Forecasting for Infectious Diseases, National Institute for Viral Disease Control and Prevention, Chinese Center for Disease Control and Prevention, Beijing 102206, China; lichenyuaaa@foxmail.com (C.L.); caoyx@ivdc.chiancdc.cn (Y.C.); 13179818951@163.com (Q.M.); yang_jing4567@126.com (J.Y.); zhangxg@ivdc.chinacdc.cn (X.Z.); lihx@ivdc.chinacdc.cn (H.L.); xuke@ivdc.chinacdc.cn (K.X.); jiangtao@ivdc.chinacdc.cn (T.J.); 2 Hebei Key Laboratory for Chronic Diseases, School of Basic Medicine Sciences, North China University of Science and Technology, Tangshan 063210, China; lsy5001@sina.com

**Keywords:** EBV, ferritin, subunit vaccine, immune response, immunogenicity

## Abstract

Considering the absence of a widely utilized EBV vaccine, we have developed an EBV gL-gH344-Ferritin nanoparticle vaccine utilizing ferritin as a carrier. The gL-gH344-Ferritin fusion gene was synthesized and inserted into the pET30a plasmid. The expression of the fusion protein in the recombinant plasmid was verified by Western blot. Then, the gL-gH344-Ferritin subunit nanoparticle vaccine was obtained by purification of the fusion protein. BALB/c mice were immunized using a two-dose protocol. The titers of EBV specific antibodies were determined using enzyme-linked immunosorbent assay at 4, 8, and 12 weeks after the initial immunization. Moreover, the levels of EBV gL-gH344 specific splenocytes secreting interferon (IFN)-γ and interleukin (IL)-6 were determined using an enzyme-linked immunospot assay. The pET30a-gL-gH344-Ferritin prokaryotic expression plasmid was successfully constructed. gL-gH344-Ferritin was efficiently expressed in *E. coli*. Following immunization with gL-gH344-Ferritin, the mice sera demonstrated elevated titers of EBV specific immunoglobulin G. Moreover, after stimulating with EBV gL-gH344 specific peptides, the splenocytes of the immunized mice showed a marked tendency to secrete large amounts of IFN-γ and IL-6. The gL-gH344-Ferritin nanoparticle vaccine carrying the EBV gL-gH344 fusion protein induced robust and sustained specific humoral and cellular immune responses in mice.

## 1. Introduction

Epstein–Barr virus (EBV), a double-stranded DNA virus belonging to the γ-herpesvirus subfamily, is one of the most common human viruses [1]. EBV infection is widespread and is primarily transmitted through saliva, with most infected individuals exhibiting asymptomatic or mild symptoms and lifelong latent infections [2]. EBV is closely associated with various diseases, including infectious mononucleosis, nasopharyngeal carcinoma, and gastric cancer, posing a significant threat to human health [3,4]. As a global pathogen, research on EBV and the current status of EBV vaccines are still in the preliminary stages, and no vaccine against EBV has been approved for marketing or widespread use [5]. Therefore, the development of a vaccine has become a critical research topic.

The gH/gL antigen is a key glycoprotein complex essential for EBV infectivity and acts as a critical protein for inducing membrane fusion [6]. EBV modulates the form of the gH/gL complex to switch infection preferences, ensuring sustained infection in B and epithelial cells [7,8]. The gH/gL complex can elicit higher neutralizing antibody titers and has a greater potential to induce immune responses, potentially providing complete protection against EBV infection [9]. Vaccine carriers are essential components of vaccines. Ferritin nanoparticles, a naturally occurring protein, have good biocompatibility and biodegradability and are stable, modifiable, and easy to produce, providing significant advantages in vaccine developments [10]. Ferritin nanoparticle-based vaccines have been proven to be novel, safe, and highly immunogenic with considerable potential [11,12]. In recent years, significant progress has been made in vaccine research against EBV. Malhi developed a self-assembled nanoparticle vaccine that demonstrated the gH/gL protein of EBV and was able to protect humanized mice against a lethal viral challenge [13]. This study provides an important reference for nanoparticle-based EBV vaccine design. In addition, a study by Edwards also demonstrated that immunization with nanoparticles displaying the gH/gL proteins of EBV resulted in limited cross-protection against rhesus lymphocyte virus [14]. These studies further reveal the potential value of gH/gL proteins in vaccine development. However, despite these research basis, the present study explored innovatively and made new findings in the use of gH truncation (gH344) for vaccine design in this study.

Therefore, the design and optimization of the EBV key antigen (gH/gL) using recombinant protein technology, combined with ferritin nanoparticle carriers, can enhance the immunogenicity and longevity of the EBV ferritin nanoparticle vaccine. This would provide a safe and efficient pathway for the designing of EBV vaccines, offer important references for the development of EBV vaccines, and provide effective solutions for the prevention and treatment of EBV-related diseases. This study aimed to develop a gL-gH344-Ferritin nanoparticle subunit vaccine comprising a fusion protein formed by linking EBV-gL to an EBV-gH truncation (gH344), with the objective of boosting immunogenicity efficacy against EBV.

## 2. Materials and Methods

### 2.1. Ethics Statement

All animal experimental procedures were approved by the Animal Care and Welfare Committee of the National Institute for Viral Disease Control and Prevention at the Chinese Center for Disease Control and Prevention (Approval No. 20241012080).

### 2.2. Construction of the Recombinant Plasmid

**Figure 1:**
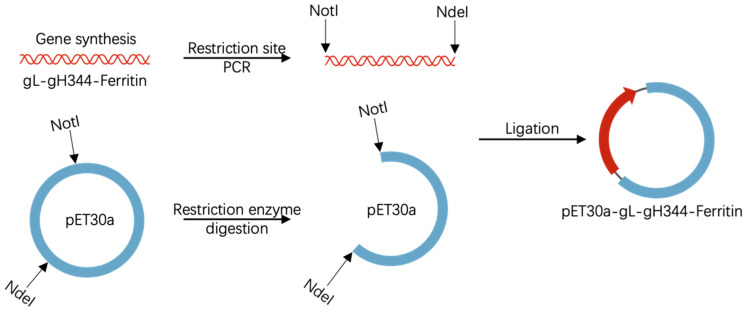
Schematic representation of the pET30a-gL-gH344-Ferritin recombinant plasmid construction. The figure includes elements adapted from BioGDP.com [15].

The gH344 subunit (a truncated variant of full-length gH lacking residues 345–679) was flanked by flexible glycine-serine linkers (GGGGS × 3) at both ends, thus connecting gL and the ferritin nanoparticle scaffold (helicobacter pylori). The recombinant plasmid pET30a-gL-gH344-Ferritin was custom-synthesized by GenScript Biotech Co., Ltd. (Nanjing, China). As shown in Figure 1, synthesized gL-gH344-Ferritin DNA fragments were PCR-amplified to introduce NotI and NdeI restriction sites at the termini, respectively. These fragments were subsequently ligated into the pET30a(+) vector (Novagen, Darmstadt, Germany), which had been pre-digested with NotI and NdeI (New England Biolabs, Ipswich, MA, USA) at 37 °C for 2 h. Ligation reactions were performed using T4 DNA ligase (Thermo Fisher Scientific, Waltham, MA USA) at 16 °C for 1 h. The ligated plasmids were then transformed into DH5α competent cells. All constructions were validated by bidirectional Sanger sequencing, confirming sequence identity to the reference design.

### 2.3. Expression and Purification of the Recombinant Proteins

Modified pET30a expression plasmids encoding the gL-gH344-Ferritin recombinant protein were individually introduced into BL21 (DE3) competent cells (TIANGEN, Beijing, China). After overnight incubation at 37 °C on Luria–Bertani (LB) agar plates supplemented with 50 μg/mL kanamycin and 33 μg/mL chloramphenicol, a single positive colony was transferred into 10 mL of LB medium with 50 μg/mL kanamycin and 33 μg/mL chloramphenicol and grown overnight at 37 °C with shaking at 220 rpm. The resulting seed culture was expanded at a 1:100 ratio until the A600 was 0.6. Isopropyl-β-D-1-thiogalactopyranoside (IPTG) was added to a final concentration of 1 mM for induction at 20 °C for 16–20 h with shaking at 150 rpm. The bacterial cultures were harvested by centrifugation at 7000 rpm and 4 °C for 20 min.

For the purification of gL-gH344-Ferritin, the cell pellets were resuspended in a lysis buffer and lysed using an ultrasonic homogenizer to break the bacterial cell walls (SCIENTZ, Ningbo, Zhejiang, China). The cell debris was removed by centrifugation at 40,000× *g* for 1 h. The supernatant was filtered using a Steritop cartridge (0.22 μm pore size) and loaded onto a gravity flow column containing Ni-NTA resin (Cytiva, Boston, MA, USA). The target protein was eluted using 50 mM Tris-HCl, 150 mM NaCl, and 10% Glycerol at pH 8.0. The collected proteins were analyzed using sodium dodecyl sulfate-polyacrylamide gel electrophoresis (SDS-PAGE) and Western blot to determine whether the target protein was collected. After concrete confirmation of the purity and yield of the protein, the fractions were pooled, concentrated, and stored at 4 °C.

### 2.4. Identification of the Recombinant Proteins

The recombinant proteins were identified using SDS-PAGE. Briefly, the purified protein was combined with an appropriate amount of 5× loading buffer, heated at 95 °C for 5 min, and separated on a 12% polyacrylamide Tris-glycine gel for 30 min at 300 V. The gels were stained with Coomassie brilliant blue (TIANGEN, Beijing, China), and de-stained with 30% methanol and 10% glacial acetic acid in double-distilled water until a clear background appeared. Recordings were conducted using a chemiluminescence instrument (Tanon, Shanghai, China).

The recombinant proteins were identified using Western blot. Briefly, the proteins were loaded onto 8% SDS-PAGE and then transferred to a polyvinylidene fluoride membrane. The membranes were probed with commercial anti-His-Tag antibodies (1:1000 dilution; Zhongshan Golden Bridge, Zhongshan, China). The horseradish peroxidase (HRP)-conjugated goat anti-mouse antibodies (1:10,000 dilution; Zhongshan Golden Bridge, China) were used as the secondary antibody. ECL chemiluminescent solution (Vazyme, Nanjing, China) was evenly dripped onto the membrane, and a recording was conducted using the chemiluminescence instrument. The color was developed using a 3,3′-diaminobenzidine kit (Zhongshan Golden Bridge, Zhongshan, China).

### 2.5. Transmission Electron Microscope Imaging

Transmission electron microscopy was performed at the National Institute for Viral Disease Control and Prevention, CDC, China. For negative staining, carbon grids were first immersed in the gL-gH344-Ferritin sample. Subsequently, the particles were stained with phosphotungstic acid. After the samples were dried, they were examined using a Tecnai 12 electron microscope (Thermo Fisher Scientific, Waltham, MA, USA).

### 2.6. Immunization and Detection Protocols for BALB/C Mice

**Figure 2:**
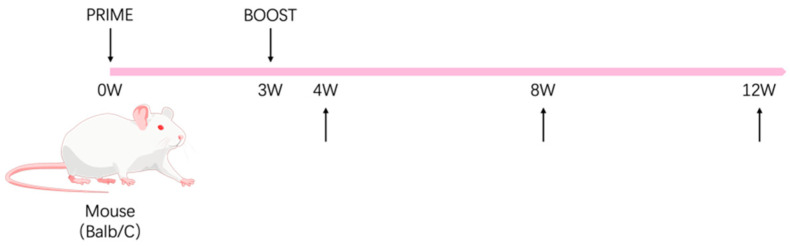
Schematic representation of the animal immunization procedure. The mouse illustration was adapted from BioGDP.com [15].

Specific pathogen-free (SPF) female BALB/c mice (aged 6–8 weeks) were purchased from Vital River Laboratories (Beijing, China) and randomly divided into three groups (*n* = 15/group). The mice were immunized intramuscularly at weeks 0 and 3. The experimental group received 15 μg/100 μL of gL-gH344-Ferritin mixed with 50% (*v*/*v*) Al hydrogel^®^ adjuvant (2% suspension, France). The control group received 2 × 10^6^ PFU/100 μL of MVA-gHgL, which was generated via homologous recombination to incorporate the full-length gHgL gene into the MVA genome. Negative controls were immunized with an equal volume of PBS. The mice were then sacrificed for immunogenicity evaluation at weeks 4, 8, and 12 after the prime vaccination, and five mice were selected from each group (Figure 2). Whole blood samples were collected using orbital hematocrit. The samples were then left at room temperature for 30 min, followed by centrifugation at 1500 rpm for 10 min. The upper layer of the serum samples was collected and stored at −20 °C for antibody detection. The mice were euthanized by cervical dislocation, and their spleens were meticulously removed and processed. Splenic lymphocytes were isolated using a mouse lymphocyte isolation solution (Dakewe, Shenzhen, China) according to the manufacturer’s instructions, and the total cell count was determined using an automated cell counter (Beckman, Brea, CA, USA).

### 2.7. Determination of Humoral Immunity in the Immunized Mice

The presence of the EBV-gHgL antibodies in the mouse serum was detected using an indirect enzyme-linked immunosorbent assay (ELISA). Briefly, an ELISA plate was coated with 100 ng/well purified EBV (strain B95-8) gH and gL heterodimer protein (His- and Flag-Tag) (Sino Biological, Beijing, China) and incubated at 4 °C overnight. The coating solution was then discarded, and the plate was covered with PBS containing 5% skimmed milk. A gradient dilution of the serum sample to be tested was then added, and the plate was incubated for 1 h and washed. A 1:20,000 dilution of HRP-labeled goat anti-mouse immunoglobulin G (IgG) antibody (Zhongshan Golden Bridge, Zhongshan, China), IgG1 (Abcam, Cambridge, UK), IgG2a (Abcam, Cambridge, UK), or IgG2b (Abcam, Cambridge, UK) was added, and the color development and termination solutions were sequentially added. The OD values at 450 nm and 630 nm were measured using an enzyme labeler (Thermo Fisher Scientific, Waltham, MA, USA). The OD value of the sample wells was found to be more than two-fold higher than that of the negative control wells, which was considered positive. The highest serum dilution that corresponds to the positive wells was determined as the antibody titer of the samples.

### 2.8. Determination of Cellular Immunity in the Immunized Mice

The mouse H^2d^ restricted EBV gHgL epitope peptides were synthesized and identified in our previous experiments [16]. These peptides were designed to mimic key epitopes of the EBV gHgL protein, aiming to activate specific immune responses. In order to assess the immune-activating effects of these peptide stimulators, the assay was performed using mouse interferon (IFN)-γ or interleukin (IL)-6 ELISpot Flex kit (Mabtech, Nacka Strand, Sweden). In summary, a mouse IFN-γ antibody was utilized to coat an enzyme-linked immunospot (ELISpot) assay plate (MabTech, Sweden) at a concentration of 75 ng/well. The plate was then subjected to overnight incubation at a temperature of 4 °C. Following the elimination of the solution, the plate was seeded with 200,000 splenocytes/well and incubated for 36 h at 37 °C, 5% CO_2_. At the same time, the test wells were supplemented with an EBV gHgL-specific peptide stimulator. The positive control wells were supplemented with the positive control (Sigma, St. Louis, MO, USA), and the control wells were supplemented with Roswell Park Memorial Institute medium (Gibco, Grand Island, NY, USA). The plates were incubated with a detection antibody at room temperature for two hours, followed by the addition of HRP-conjugated streptavidin (1:1000) and further incubation for one hour at ambient temperature. Following a thorough washing step with PBST, the plates were then supplemented with TMB substrates and developed until the emergence of distinct spots was observed. The reaction was terminated by subjecting the mixture to extensive washing with deionized water. The number of spots was determined and analyzed using an ImmunoSpot S6 analyzer (Cellular Technology Limited, Shaker Heights, OH, USA).

### 2.9. Statistical Analysis

The level of humoral immunity was represented by the geometric mean of the antibody titers against EBV-gHgL in the serum of mice at the time of detection. The intensity of the specific cellular immune response was indicated by the average number of EBV-gHgL-specific INF-γ spot-forming cells per million splenic lymphocytes (spot-forming cells (SFCs)/10^6^ lymphocytes). Statistical analysis were performed using GraphPad Prism 7.0 software (GraphPad Software, San Diego, CA, USA) and the differences between the experimental and control groups at each time point were compared using two-way ANOVA. The level of statistical significance was set at *p* < 0.05.

## 3. Results

### 3.1. Design and Expression of the pET30a-gL-gH344-Ferritin Recombinant Plasmid

**Figure 3:**
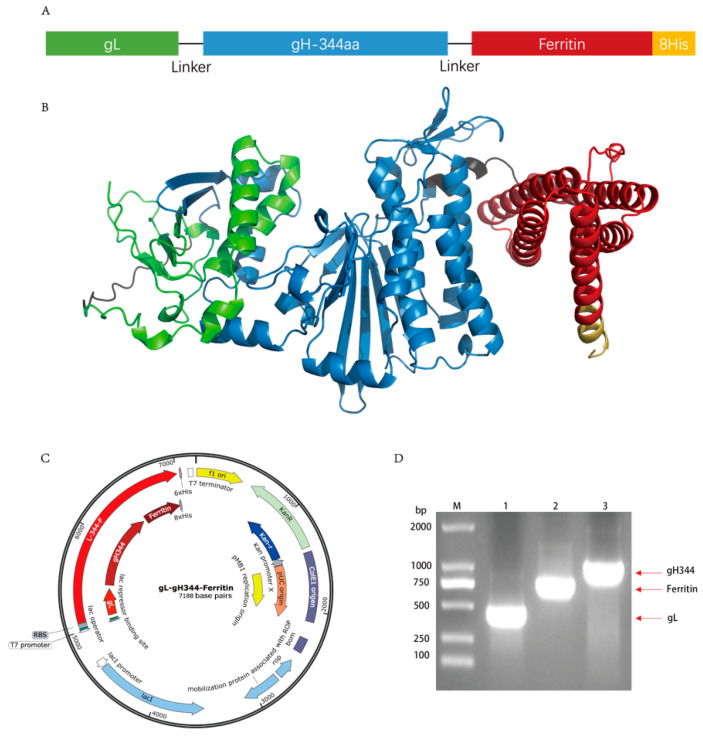
Design of the gL-gH344-Ferritin recombinant protein. (**A**) schematic representation of the gL-gH344-Ferritin recombinant protein. (**B**) predicted three-dimensional structure of the gL-gH344-Ferritin recombinant protein. The structure was modeled using AlphaFold. (**C**) pET30a-gL-gH344-Ferritin plasmid map generated by SnapGene. (**D**) identification of the pET30a-gL-gH344-Ferritin recombinant plasmid. M: DNA Marker; 1: gL-F/R amplified product; 2: Ferritin-F/R amplified product; 3: gH344-F/R amplified product.

The extracellular region of the gH protein comprises four major structural domains, with Domain I (D-I) and Domain II (D-II) being critical for the surface expression and cellular entry functions of gH [17,18]. Notably, the D-II region harbors the predominant antigenic peptide epitopes of the gH gene [16]. Based on these findings, gH344, a truncated variant of gH was fused with gL via a flexible linker to facilitate the formation of a functional fusion protein. This construct was further engineered to link with ferritin nanoparticles and serve as a vaccine vector (Figure 3A). The protein structure was predicted using AlphaFold (Figure 3B). Figure 3C shows the SnapGene generation map of the pET30a-gL-gH344-Ferritin plasmid, illustrating the arrangement of the inserted genes and key features of the construct, which was essential for confirming the successful cloning. The plasmid was amplified by PCR, and the product was analyzed using 1% agarose gel electrophoresis, confirming the presence of specific fragments corresponding to gL (453 bp), ferritin (765 bp), and gH344 (1008 bp) of the expected sizes (Figure 3D). The insertion was further verified by Sanger sequencing.

### 3.2. Expression and Characterization of the gL-gH344-Ferritin Recombinant Protein

**Figure 4:**
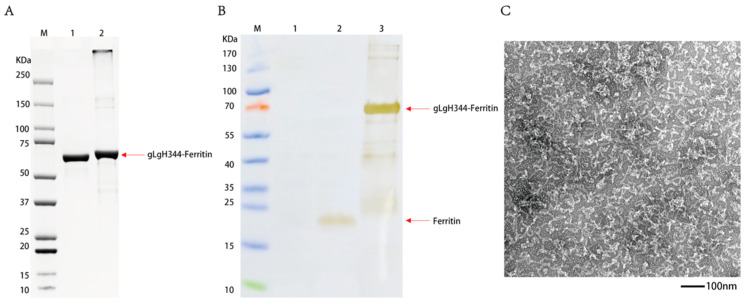
Identification of the gL-gH344-Ferritin recombinant protein. (**A**) sodium dodecyl sulfate-polyacrylamide gel electrophoresis (SDS-PAGE) identification of the gL-gH344-Ferritin recombinant protein: Lane M, protein marker; lane 1, 2 μg BSA; and lane 2, gL-gH344-Ferritin protein. (**B**) Western blot identification of the gL-gH344-Ferritin recombinant protein: Lane M, protein marker; lane 1, BL21 cell lysate; lane 2, his-tagged ferritin protein; and lane 3, gL-gH344-Ferritin protein. (**C**) TEM imaging of the gL-gH344-Ferritin recombinant protein.

The expression plasmid encoding pET30a-gL-gH344-Ferritin was transformed into *E. coli* BL21(DE3) for IPTG-induced expression and subsequently purified via nickel column affinity chromatography. The resulting protein samples were analyzed by SDS-PAGE, with the results shown in Figure 4A. gL-gH344-Ferritin was successfully expressed in *E. coli* BL21(DE3), exhibiting the expected molecular weight of approximately 70.9 kDa. Furthermore, the protein was produced in substantial quantities under induced conditions. Western blot was performed to further confirm the identity of the protein. As shown in Figure 4B, the specific bands aligned with the expected size of gL-gH344-Ferritin (70.9 kDa), confirming the presence of the protein in the purified samples. The His-Tag specifically recognized gL-gH344-Ferritin, validating its successful expression and purification. The protein was concentrated, and its concentration was determined to be 0.42 mg/mL using the Bradford method. Figure 4C shows the negative staining transmission electron microscopy (TEM) image of the gL-gH344-Ferritin recombinant protein. The expected icosahedral cage-like structure was not formed. This is likely due to the excessive length of gL-gH344, which may have hindered the proper assembly of the cage-like structure. However, the resulting amorphous structure might still be functional and could potentially play a role in the study.

### 3.3. Specific Antibody Levels Induced by the gL-gH344-Ferritin Vaccine

BALB/c mice were immunized at weeks 0 and 3, while the negative control group received PBS. Serum samples were collected at 4, 8, and 12 weeks post-primary immunization (w.p.i.) to measure EBV-gH/gL antibody titers (Figure 2). The recombinant gH/gL fusion protein, produced in a mammalian expression system, was used as an antigen. This protein closely mimics the native protein in conformation and glycosylation, ensuring accurate antibody titer assessment.

**Figure 5:**
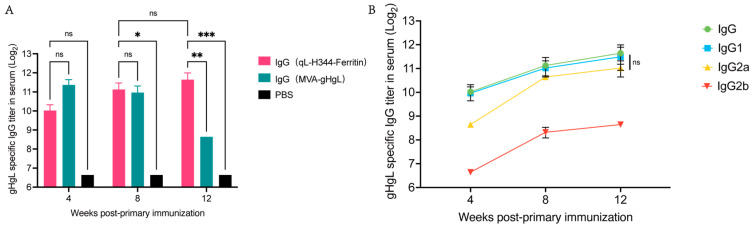
Serum-specific antibody levels induced by the gL-gH344-Ferritin and MVA-gHgL vaccines in mice. (**A**) titers of specific immunoglobulin G (IgG) antibodies induced by the gL-gH344-Ferritin and MVA-gHgL vaccines as determined using enzyme-linked immunosorbent assay (ELISA); * *p* < 0.05; ** *p* < 0.01; *** *p* < 0.001; ns indicates no significant difference. (**B**) titers of specific IgG, IgG1, IgG2a, and IgG2b antibodies induced by the gL-gH344-Ferritin vaccines as determined using ELISA. Statistical analysis was performed using two-way ANOVA to evaluate significant differences. ns indicates no significant difference.

Following the completion of the immunization procedures, we proceeded to evaluate the IgG titers in the serum of the immunized mice. As depicted in Figure 5A, both the gL-gH344-Ferritin and MVA-gHgL vaccinated groups displayed detectable levels of specific antibodies at 4, 8, and 12 weeks post-primary immunization (w.p.i), when compared to the PBS control group. This finding indicates that a sustained immune response was induced in the vaccinated mice. It is noteworthy that at 4 and 8 weeks there was no significant difference in IgG titers between the gL-gH344-Ferritin and MVA-gHgL groups. However, at 12 weeks, the gL-gH344-Ferritin vaccinated group showed a marked advantage, with higher IgG titers, suggesting a more robust immune response at this later time point.

Subsequently, we analyzed the titers of various IgG subclasses, as shown in Figure 5B. The ELISA results demonstrated that both IgG1 and IgG2a titers increased over time, exhibiting similar trends for both subclasses. Additionally, IgG2b titers also displayed an upward trend. Importantly, there were no statistically significant differences observed between IgG1 and IgG2a titers. These findings suggest that the gL-gH344-Ferritin vaccine induced a balanced Th1/Th2 response, without favoring either type.

### 3.4. Expression of Cellular Immunity Induced by the gL-gH344-Ferritin Vaccine

**Figure 6:**
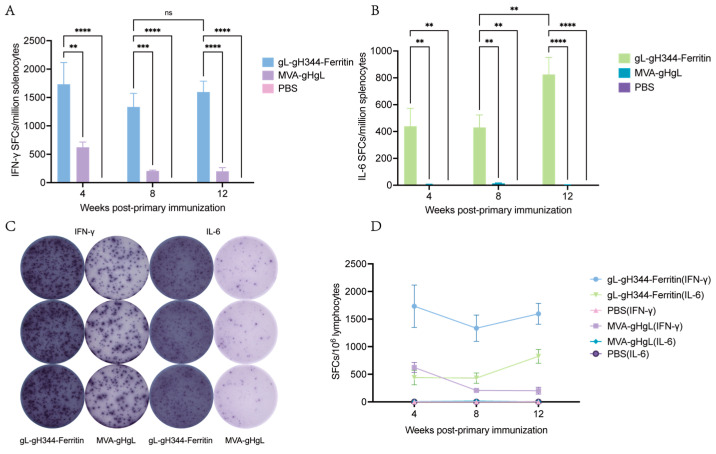
Induction of EBV-gH/gL-specific cellular immunity by the gL-gH344-Ferritin and MVA-gHgL vaccines in mouse splenic lymphocytes. (**A**) levels of interferon (IFN)-γ secretion from mouse splenic lymphocytes induced by the gL-gH344-Ferritin and MVA-gHgL vaccines, as determined using enzyme-linked immunospot (ELISpot) assay. (**B**) levels of interleukin (IL)-6 secretion from mouse splenic lymphocytes induced by the gL-gH344-Ferritin and MVA-gHgL vaccines, as determined using ELISpot assay. Data were analyzed using two-way ANOVA to evaluate significant differences. ** *p* < 0.01; *** *p* < 0.001; **** *p* < 0.0001; ns indicates no significant difference. (**C**) representative ELISpot images showing IFN-γ or IL-6 secretion from mouse splenic lymphocytes induced by the gL-gH344-Ferritin and MVA-gHgL vaccines. (**D**) weekly comparison of IFN-γ and IL-6 levels among the gL-gH344-Ferritin, MVA-gHgL, and PBS control groups.

As illustrated in Figure 6A,C, the gL-gH344-Ferritin vaccine induced a pronounced IFN-γ response characterized by 1733 IFN-γ- spots forming cells (SFCs) per million splenocytes at 4 w.p.i. This response showed a moderate attenuation at 8 w.p.i. and 12 w.p.i. (remaining > 1300 SFCs), while IL-6 secretion exhibited a distinct kinetic pattern: initial levels remained below 500 SFCs during 0–8 w.p.i., followed by a significant surge to 825 SFCs by 12 w.p.i. (Figure 6B,C). Critically, sustained IFN-γ dominance did not correlate with suppression of IL-6 production (Figure 6D), suggesting a coordinated rather than polarized Th1/Th2 immune modulation.

## 4. Discussion

The gL-gH344-Ferritin vaccine, designed by displaying the gH/gL complex on ferritin nanoparticles, synergistically combined the immunogenicity of the gH/gL antigenic complex with the delivery efficiency of the ferritin nanoparticle platform. This innovative approach exploited the unique structural attributes of ferritin to enhance its antigen presentation and immune activation. Our findings demonstrated that the gL-gH344-Ferritin nanoparticle vaccine robustly induced both humoral and cellular immune responses. This vaccination strategy holds promise for the development of an effective prophylactic intervention against EBV infection.

gH344, a truncated variant of gH, retains the integral D-I and D-II regions. Notably, the D-II region encompasses two disulfide bonds that are crucial for the cell surface expression of the gH/gL complex and to modulate the fusion capacity with B and epithelial cells [19]. Moreover, the D-II region contains the primary antigenic peptide epitope of gH, which effectively activates both cellular and humoral immune responses [16]. The gH344 domain retains these critical epitopes, ensuring robust immune responses. Additionally, it offers enhanced stability and reduced complexity in vaccine design [16]. The gH and gL glycoproteins form a heterodimeric complex indispensable for viral cell entry, efficient membrane fusion, and direct engagement with epithelial cell receptors [20]. Given these functional and immunogenic attributes, the combination of gH344 and gL has emerged as a promising vaccine epitope candidate, offering a strategic approach to combat EBV infections. The gL-gH344-Ferritin vaccine demonstrated robust immunogenicity, as evidenced by its ability to elicit high titers of IgG antibodies specific to the gH/gL complex. These antibodies are widely recognized to mediate protective immunity against the EBV infection [21]. Such potent antibody induction is critical for enhancing host resistance to EBV [21,22].

The expected icosahedral cage-like structure of the gL-gH344-Ferritin recombinant protein was not observed under the transmission electron microscopy (Figure 4C). However, our functional assays demonstrated that the gL-gH344-Ferritin fusion protein elicited robust and sustained immune responses in mice, particularly cellular immune responses. This suggests that despite the structural differences, the fusion protein effectively activates T cells, contributing to a strong adaptive immune response.

Immune responses can be categorized into Th1- or Th2-dominated types based on cytokine profiles [23]. Th1 cells combat intracellular pathogens via cell-mediated immunity, primarily through IFN-γ secretion [24]. This cytokine activates macrophages and cytotoxic T cells while promoting B cell class switching to opsonizing IgG2a antibodies [24]. In contrast, Th2 cells defend against extracellular pathogens by orchestrating humoral immunity through cytokines (e.g., IL-4, IL-5, IL-6) [25]. These cytokines drive eosinophil recruitment and mast cell activation while also promoting IgG1 antibody production, which is critical for neutralizing parasites [26].

To characterize the immune response induced by the gL-gH344-Ferritin vaccine, we quantified serum IgG1 and IgG2a levels in vaccinated mice using ELISA. The results showed no significant difference between IgG1 and IgG2a titers, indicating a balanced Th1/Th2 response. This balanced immunity is advantageous for EBV protection, as it avoids an overemphasis on either cell-mediated (Th1) or antibody-dependent (Th2) responses, potentially enabling a comprehensive defense against viral entry and replication [27]. Given the high levels of binding antibodies observed, we further assessed the levels of neutralizing antibodies to evaluate the vaccine’s efficacy in inhibiting viral infection. However, due to the limited volume of mouse serum available, we pooled the serum from 8 w.p.i. mice immunized with the gL-gH344-Ferritin vaccine. The neutralizing activity against the CNE2-EBV strain of the serum pool was evaluated, with neutralization percentages of 59.42% and 43.54% being achieved at 1:10 and 1:20 dilutions, respectively. These findings suggest that EBV-specific neutralizing antibodies were induced in mice, albeit with a relatively low titer. Future studies may explore the use of combined vaccine immunization to enhance the level of neutralizing antibodies.

Further analysis of T helper cell polarization dynamics revealed unique features of the ferritin-based vaccine. We observed strong early immunogenicity, with IFN-γ responses reaching 1733 SFCs/million splenocytes by week 4, and these levels were maintained through week 12 (>85% retention). In contrast, IL-6 secretion showed a delayed but significant increase, reaching 825 SFCs by week 12 (Figure 6B). This pattern of cytokine kinetics differs from that seen with traditional Th1-dominant vaccines, such as those adjuvanted with CpG, where a surge in IFN-γ typically suppresses Th2-associated cytokines like IL-4 and IL-6 [28].The Th1/Th2 co-activation, which we observed, aligns with emerging strategies for combating complex herpesvirus infections [29]. EBV has a two-phase life cycle—initial lytic replication followed by B cell immortalization—which demands a combination of immune responses: sustained IFN-γ for viral clearance and regulated IL-6 for tissue homeostasis [30,31]. Our data suggest that the gH/gL-ferritin vaccine may effectively engage CD4+ T cell subsets, which is particularly important for preventing infectious mononucleosis, where overly polarized Th1 responses can exacerbate immunopathology [32].

The immunomodulatory profile and translational potential of the gL-gH344-Ferritin platform are further highlighted through systematic comparison with leading EBV vaccine candidates (Table 1). While conventional platforms—viral vectors (Th1-polarized with inherent vector immunity) and peptide/mRNA vaccines (constrained by linear epitopes or transient expression)—face trade-offs between immune breadth and clinical safety, our ferritin-based design reconciles these conflicts [33]. Firstly, in terms of structural vaccinology, the truncated gL-gH344 fusion retains epitopes critical for EBV entry inhibition, surpassing fragmented antigens in NCT00078494 and NCT01094405 (ClinicalTrials.gov). Secondly, regarding dynamic immune coordination, IgG1/IgG2a parity contrasts with Th1-skewed mRNA vaccines (NCT05714748), while phased IFN-γ/IL-6 synergy may address both lytic suppression (via cytotoxic T-cells) and latency control (via B-cell modulation) [34]. This dual-phase targeting strategy positions gL-gH344-Ferritin as a versatile candidate across EBV-associated pathologies [13,35].

The gL-gH344-Ferritin vaccine demonstrates advantages in cellular immunogenicity over current herpesvirus subunit vaccines. Unlike Shingrix—a licensed VZV subunit vaccine utilizing glycoprotein E and the AS01B adjuvant—our candidate has higher IFN-γ+ cell frequencies in murine models [36]. Crucially, these responses persisted at elevated levels through 12 weeks, contrasting with the transient IFN-γ activity reported for Shingrix in preclinical studies. This durability aligns with the functional demands of EBV immunity, where sustained T-cell surveillance is critical to control latent reservoirs and prevent reactivation [37].

As a recombinant protein vaccine, the gL-gH344-Ferritin nanoparticle vaccine is characterized by its ease of large-scale production in *E. coli* and a high safety profile [38]. During the immunization process in mice, no adverse reactions, such as changes in body weight, activity, or fur conditions, were observed. No scarring or ulceration was observed at the injection site. The safety profile of the gL-gH344-Ferritin nanoparticle vaccine offers broad prospects for its application in biomedicine.

## 5. Conclusions

Currently, no vaccine against EBV has been approved for marketing or widespread use, highlighting the urgent need for the development of an effective EBV vaccine. The gL-gH344-Ferritin nanoparticle vaccine is a promising candidate for an anti-EBV vaccine capable of inducing robust humoral and cellular immune responses. However, further in-depth studies are required to investigate the safety and characteristics of the induced immune responses. The gL-gH344-Ferritin nanoparticle vaccine provides a preclinical model for the development of vaccines against EBV and offers a methodological approach for constructing vaccines against other herpesviruses, potentially facilitating further research into the functional mechanisms of gH/gL.

## Figures and Tables

**Table 1:** Comparative Analysis of gL-gH344-Ferritin Vaccine with Other EBV Vaccine Candidates.

Parameter	Platform	Antigen Design	Immune Response	Key Strength	Limitations
gL-gH344-Ferritin vaccine	Ferritin nanoparticle	Full-length gL and the truncated form of gH (gH344)	Balanced Th1/Th2 (IgG1 ≈ IgG2a)	Balanced immune activation	Preclinical data only
Viral Vector Vaccine (NCT01094405)	Adenovirus	LMP1 fragment	Th1-biased (High IFN-γ, low IL-6)	Phase II efficacy validated	Vector immunity risk
Peptide Vaccine (NCT00078494)	Synthetic peptide	EBNA1 peptide	Th2-biased (High IgG1, weak IFN-γ)	Excellent safety profile	Narrow antigen coverage
mRNA Vaccine (NCT05714748)	LNP-encapsulated mRNA	undisclosed	Th1-skewed (IFN-γ dominant)	High scalability	Unknown durability of response

## Data Availability

All data related to this study are included in this article.

## Abbreviations

The following abbreviations are used in this manuscript:

EBV	Epstein–Barr virus
IFN-γ	interferon -γ
IL-6	Interlekin-6
SDS-PAGE	sodium dodecyl sulfate-polyacrylamide gel electrophoresis
Domain	D
ELISA	enzyme-linked immunosorbent assay
ELISpot	enzyme-linked immunospot
TEM	transmission electron microscopy
SFCs	spots forming cells
w.p.i.	weeks post-primary immunization
Th	T helper
